# Real-time tracking of a diffuse reflectance spectroscopy probe used to aid histological validation of margin assessment in upper gastrointestinal cancer resection surgery

**DOI:** 10.1117/1.JBO.27.2.025001

**Published:** 2022-02-01

**Authors:** Ioannis Gkouzionis, Scarlet Nazarian, Michal Kawka, Ara Darzi, Nisha Patel, Christopher J. Peters, Daniel S. Elson

**Affiliations:** a Imperial College London, Department of Surgery and Cancer, London, United Kingdom; b Imperial College London, Hamlyn Centre, London, United Kingdom

**Keywords:** diffuse reflectance spectroscopy, probe tracking, margin delineation, tissue classification, machine learning, cancer

## Abstract

**Significance:**

Diffuse reflectance spectroscopy (DRS) allows discrimination of tissue type. Its application is limited by the inability to mark the scanned tissue and the lack of real-time measurements.

**Aim:**

This study aimed to develop a real-time tracking system to enable localization of a DRS probe to aid the classification of tumor and non-tumor tissue.

**Approach:**

A green-colored marker attached to the DRS probe was detected using hue-saturation-value (HSV) segmentation. A live, augmented view of tracked optical biopsy sites was recorded in real time. Supervised classifiers were evaluated in terms of sensitivity, specificity, and overall accuracy. A developed software was used for data collection, processing, and statistical analysis.

**Results:**

The measured root mean square error (RMSE) of DRS probe tip tracking was 1.18±0.58  mm and 1.05±0.28  mm for the x and y dimensions, respectively. The diagnostic accuracy of the system to classify tumor and non-tumor tissue in real time was 94% for stomach and 96% for the esophagus.

**Conclusions:**

We have successfully developed a real-time tracking and classification system for a DRS probe. When used on stomach and esophageal tissue for tumor detection, the accuracy derived demonstrates the strength and clinical value of the technique to aid margin assessment in cancer resection surgery.

## 1 Introduction

Cancers of the gastrointestinal (GI) tract remain a major contributor to the global cancer risk, with ∼4.8 million new cases of GI cancer worldwide in 2018.^1^ These malignancies continue to pose a major threat to public health. The aim of surgery is for complete resection of tumor with clear margins, while preserving as much surrounding healthy tissue as possible.^2^ A positive circumferential resection margin (CRM) is associated with local recurrence of the tumor and poorer long-term survival.^3^^,^^4^ The five-year survival of patients with esophageal cancer with positive margins has been reported as 13.8% compared to 46.3% for those with negative margins.^5^ In patients with stomach cancer, local recurrence has been shown to be 16% after a positive margin following surgery.^6^ For this reason, it is paramount to establish tissue margins accurately.^7^

Currently, the gold-standard intra-operative technique for CRM assessment is frozen sections.^8^^,^^9^ However, this technique is at risk of sampling errors, plus it is time-consuming, labor intensive, and lengthens the operative time, affecting both patient outcome and theater efficiency.^10^

Diffuse reflectance spectroscopy (DRS) is a technique that allows discrimination of normal and abnormal tissue based on spectral data and presents a promising advancement in cancer diagnosis.^11^^–^^14^ When compared to sophisticated micro-endoscopic probes, DRS has lower costs and is simpler because it does not require lasers or magnification optics. The main limitation of the clinical use of DRS is that, although DRS can discriminate tissue types, it does so by providing single-point spectral measurements and leaves no marks on the tissue during scanning.^15^ In this way, it is not possible to localize the area that has been in contact with the probe when optical biopsy takes place, and thus makes it difficult for the surgeon to determine the resection margin. This is particularly challenging when DRS is used endoscopically or during minimally invasive surgery, where the ergonomics of scanning and viewing the DRS probe site are even more demanding.

To overcome this limitation, the tip of the DRS probe should ideally be tracked to enable localization of the biopsy site.^16^ Several surgical tool tracking approaches are available, of which optical tracking has been the most widely evaluated during surgical practice.^17^^,^^18^ This involves using color images from one or more cameras to determine a tool’s pose and has been particularly used during minimally invasive surgery.^19^ Many optical tracking systems involve markers such as fiducials or color labels such that the tip of the instrument can be recognized and tracked. For instance, a dual-pattern hybrid marker, incorporating both circular dots and chessboard vertices, provided high detection rates and accurate pose estimation of a laparoscopic gamma probe achieving a mean translation error of 1.81 mm.^20^ In another study, biocompatible color markers appropriate for real-time use during surgery achieved 75% sensitivity and 90% specificity for a multiple instrument tracking system.^21^

Other studies have developed systems to delineate resection margins; however, they have been met by significant limitations. Raman spectroscopy has shown much promise in this field, but it has not yet been possible to perform probe tracking in real time, although a rate of 2 to 5 frames per second has been achieved.^22^ Similarly, although fluorescence-guided surgery has the potential for real-time margin assessment, quantifiable fluorescence imaging information is difficult to obtain using open-field devices due to uncontrollable parameters, such as the variable light intensity, working distance, and influence of ambient light on the instruments in the operating room.^23^

This paper presents a tracking system to enable localization of the tip of a handheld DRS probe. The system allowed tracking of the two-dimensional (2D) position and orientation (x and y axes, angle) of the spectroscopic probe using a color marker to aid real-time assessment of tumor margins when used on *ex vivo* cancer specimens. The aim of this study was to support real-time differentiation of tumor and non-tumor tissue—and thereby to improve existing methods for intra-operative margin assessment—by introducing a system to track a DRS probe during surgery.

## 2 Methods

### 2.1 Marker Design, Detection and Segmentation, and Calculation of Tip Position

We first describe the tracking system for the DRS probe, with details about the probe itself and the *ex vivo* experiments presented in later sections. Accurate detection and segmentation are essential for DRS probe tracking. To calculate the 2D probe tip position, we needed to find the location of the probe in the image plane. The midline of the DRS probe could be found from the two edges, with the tip point lying somewhere along this line.

**Fig. 1:**
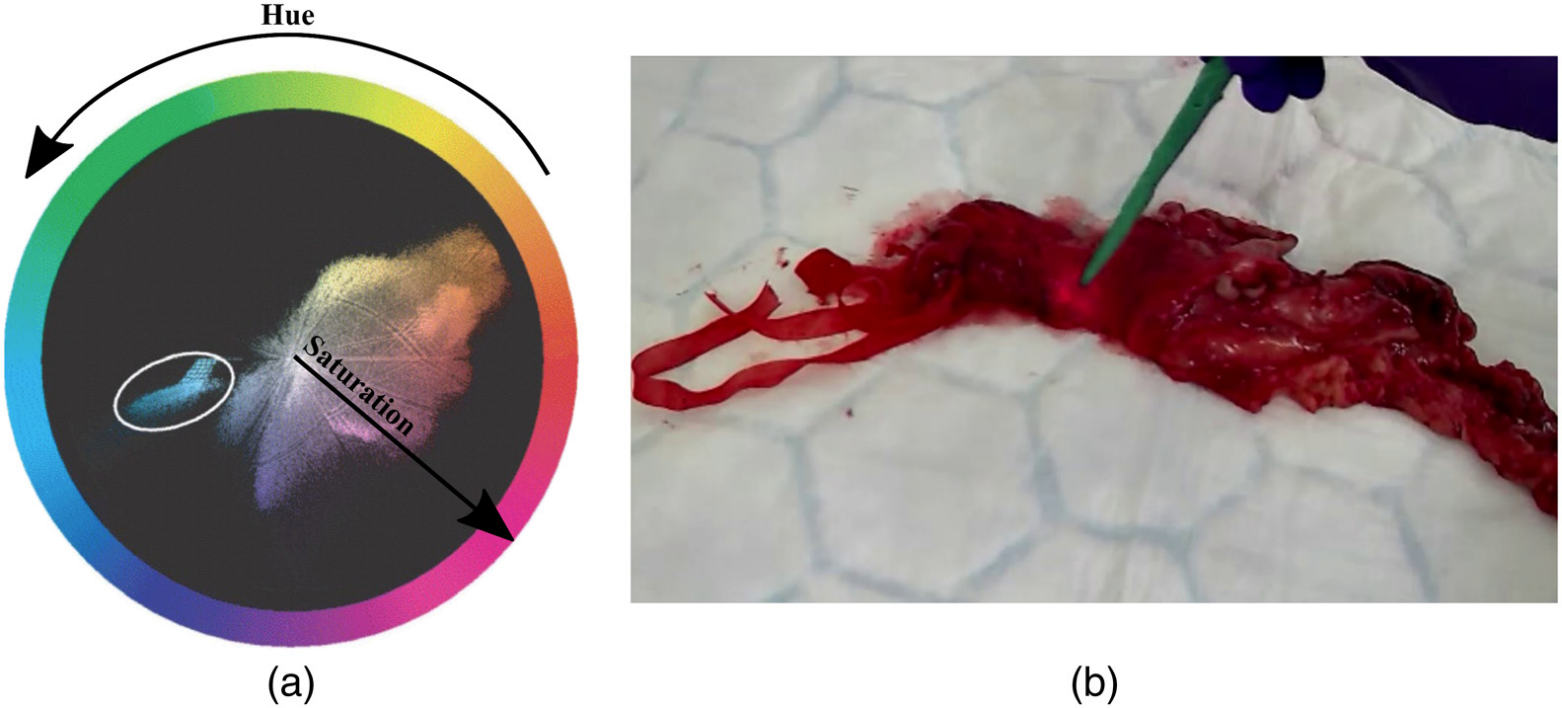
(a) Color distribution of the biological tissue, with cyan color circled as a potential color for our marker. (b) DRS fiber probe with the green color marker attached.

The non-uniqueness of the probe color inspired the use of an artificial color marker to identify it. To choose a distinct marker color, we analyzed the color distribution of real laparoscopic images that contained all possible colors that may be present during the course of an operation. The hue-saturation-value (HSV) color space (Fig. 1) was used since the hue values are directly related to the color signature. Figure 1(a) shows a typical color distribution for biological tissue ranging from yellow to purple. For the coded color to be most distinguishable from those present in the image, two alternative colors were chosen—cyan and green—to construct a marker in the form of plastic tape, 70 mm in length, wrapped close to the tip of the cylindrical probe (Fig. 1).

**Fig. 2:**
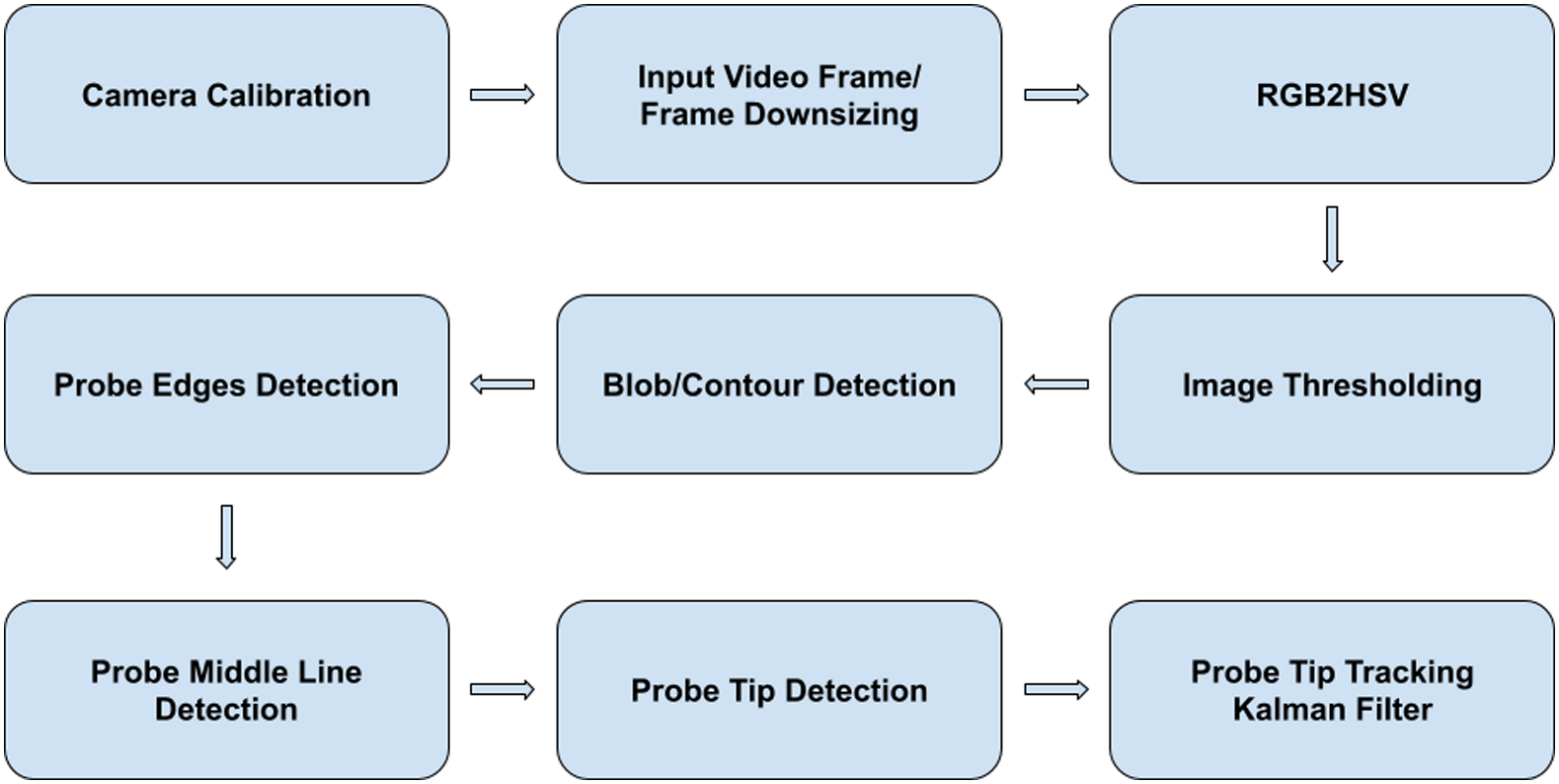
DRS fiber probe tracking procedure.

The overall methodology for using a monocular red/green/blue (RGB) camera (C920, Logitech International S.A., Lausanne, Switzerland) in a 2D color-based optical marker tracking system is summarized in Fig. 2. A 2D image calibration method was employed^24^ that only requires the observation of a planar pattern (a checkerboard) from different orientations and positions. A set of pre-captured images from the camera were analyzed to estimate the 3×3 intrinsic matrix K that contained the intrinsic parameters (e.g., focal length, optical center, and radial distortion coefficients of the lens) and the extrinsic matrix that consisted of the 3×3 rotation matrix, R, and the 3×1 translation vector.

The input video frames were resized from 1920×1080 to 640×480 for faster processing and an increase in frames-per-second. Then, the frame was blurred to reduce high spatial frequency information while maintaining visibility of the flat-textured DRS probe. The image was converted from RGB to HSV color space, and thresholded based on predefined hue (h) and saturation (s) upper and lower boundaries for either cyan or green color. The segmented frame was converted back to RGB, and morphological erosion and dilation operations were applied to eliminate negative impulsive noise and positive noise, and to fill possible gaps in the mask.

The various contours in the mask representing segmented objects were identified and ordered, and the largest one was retained as the likely position of the detected color marker. Corner points of the bounding box of the contour were extracted and used alongside the Hough transform to detect the edges of the probe. The midline of the probe was calculated from the detected edges using standard description of a line located in x–y plane: y=mx+b.

**Fig. 3:**
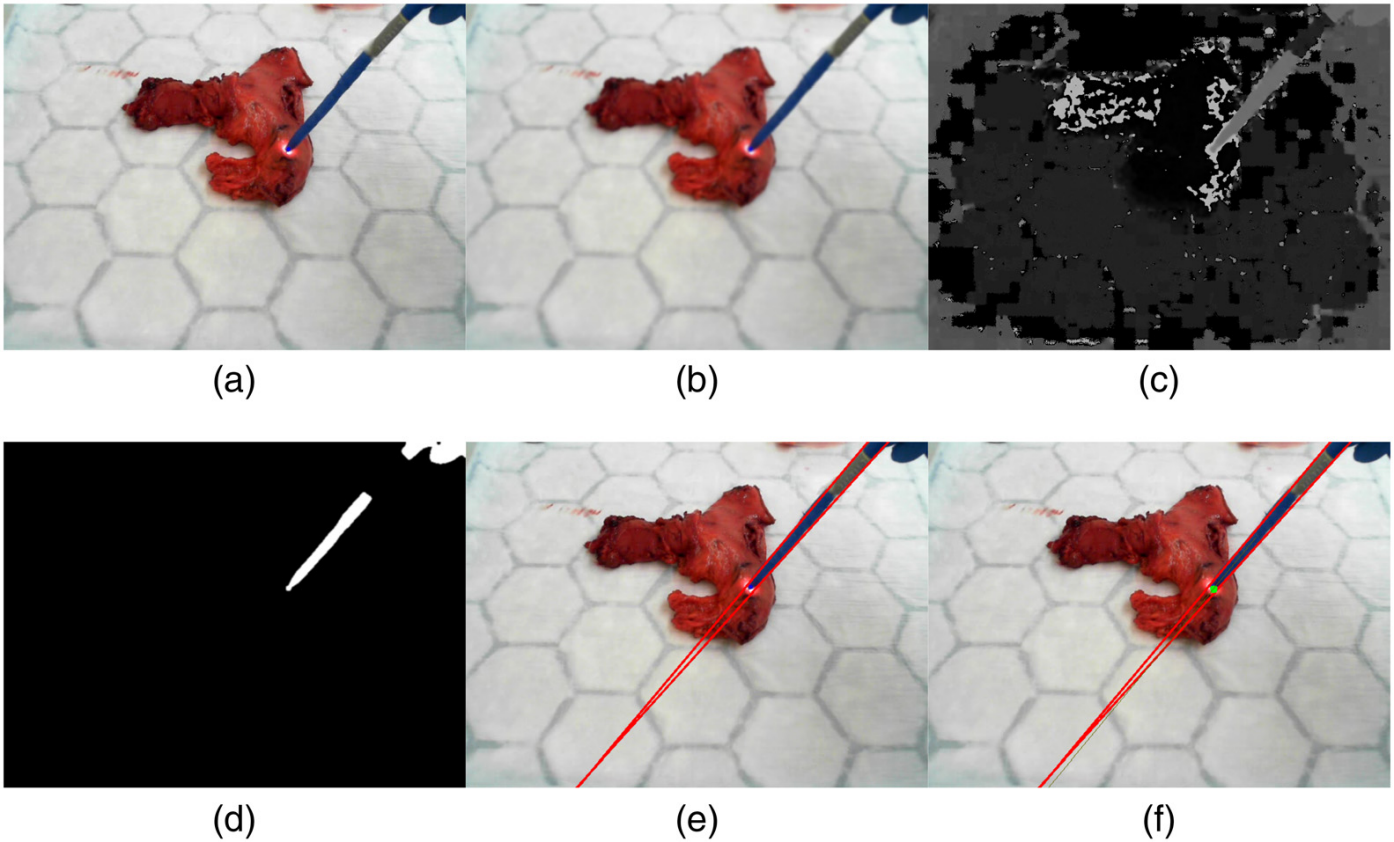
DRS probe detection workflow. (a) Example input video frame. (b) Blurred frame. (c) Hue channel of the blurred input frame. (d) Binary mask result of HSV segmentation. (e) Detected edge lines of the probe. (f) Detected tip point of the probe.

The tip position of the probe was located by conducting a one-dimensional (1D) edge detection along the midline of the probe in the segmented image. From Fig. 3(d) it is clear that there are two possible locations that could correspond to the tip position, one located to the bottom left of the white segmented probe, and the other one to the top right. To determine which direction to search for the location of the probe tip along the midline, it was assumed that the probe would only be moved in the range 0 deg to 180 deg in the yaw axis during *ex vivo* benchtop sampling.

### 2.2 DRS Probe Tip Tracking

To deal with real-world situations such as partial occlusion, changing illumination, probe velocity changes etc., we introduced a standard Kalman filter (KF) to estimate the location of the tip of the probe based on its location in previous frames.^25^ The task of the KF was divided into two steps: prediction and correction. The KF tracked the state matrix that contained the current value of the probe tip location, while the process covariance matrix contained the predictive error of those measurements. For each new time frame, the state transition matrix moved the state and process matrices based on the position at that time, estimating a new position and new covariance. The Kalman gain was calculated along with the detected probe tip positions. The correction step consisted of the update from the current state using the Kalman in conjunction with the measurement at that time frame.

### 2.3 Graphical User Interface

**Fig. 4:**
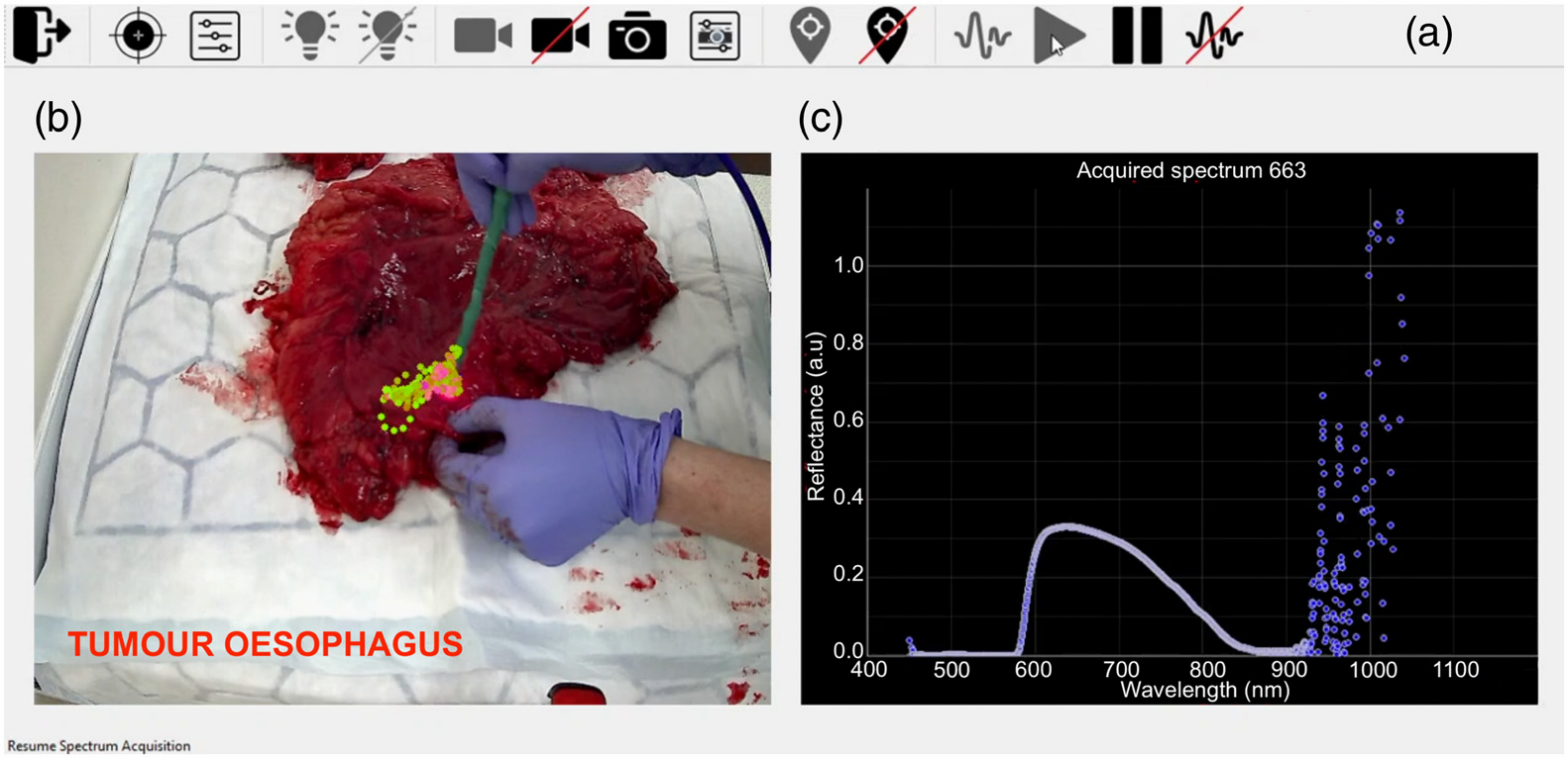
Python software for hardware system control, data acquisition and real-time classification. (a) Control panel, allowing for execution of data acquisition protocol, probe tracking, hardware calibration and light source control. (b) Augmented live video feed from the RGB camera. Real-time classification is displayed on the screen via text and a colored overlay on the tissue, with a graduated color scale indicating the probability of belonging to a particular class (Normal = 100% green – 100% pink = Tumor). (c) Reflectance data in the 420- to 1000-nm spectral range displayed in real-time.

A user interface (UI) was developed using Python 3.6 (Python Software Foundation, Wilmington) and Qt5 toolkit to integrate all the functionalities (Fig. 4) on a standard PC (Intel i3 processor at 3.30 GHz with 8 GB RAM). The UI consists of different functions such as the “start tracking” function for the initiation of the probe tracking and the “get spectrum” for the spectral data acquisition. Before starting the acquisition, the user entered patient metadata, spectrometer integration time, spectral range, tissue type, and DRS calibration etc. in a dialog box. A single frame was captured of the tissue sample being examined without the DRS probe. This frame was annotated with all the probed locations at the end of the acquisition process. The live video feedback from the RGB camera was annotated in real time with the probed positions, and the current spectrum at each location was displayed in the main GUI window. A colormap was utilized to visually indicate the probability of a data point belonging to a class of tissue type (green for normal, pink for tumor, classification described below). Real-time classification was added to the GUI following the interim analysis of the data. When the experiment was finished, all data was saved for further correlation with the histological data. The system worked at 30 frames per second with an image resolution of 640 × 480 pixels using a laptop with code parallelization. Finally, the DRS probe was able to acquire 80 spectra per second through the developed GUI using multi-threading.

### 2.4 *Ex Vivo* Data Acquisition

The study was performed with approval from the Harrow Research Ethics Committee (ref. no. 08/H0719/37) and was undertaken at Imperial College NHS trust. We collected data from patients undergoing upper GI cancer resection surgery between July 2020 and March 2021 who gave their written consent to partake in the study.

**Fig. 5:**
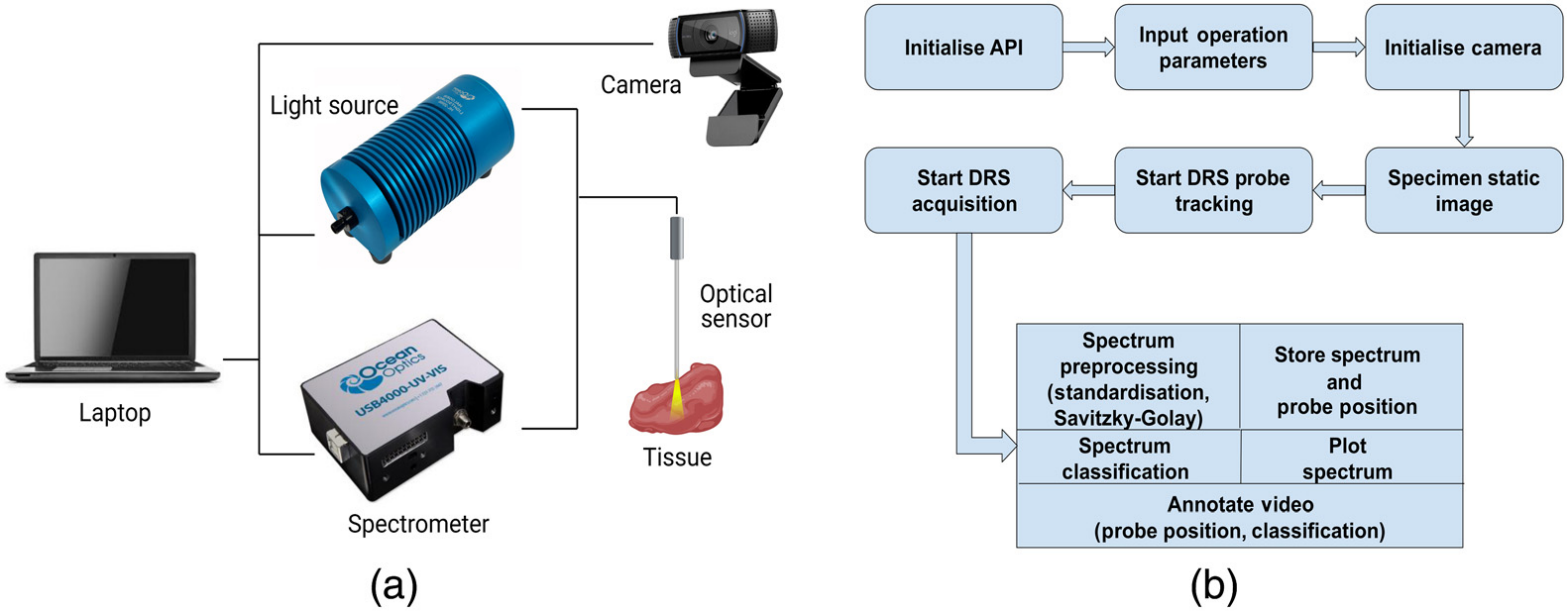
(a) DRS instrumentation and (b) workflow for *ex vivo* data acquisition.

The DRS system consisted of a reflection probe (Ocean Optics Inc., QR600-7-SR-125F) containing six peripheral illumination fibers around one light collection fiber (600  μm diameter) within a 0.125-in. ferrule. A tungsten halogen light source (360 to 2400 nm, typical output power 8.8 W, Ocean Optics Inc., HL-2000-HP) was connected to the six illumination fibers, while the central fiber was connected to a spectrometer (Ocean Optics Inc., USB4000) [Fig. 5(a)]. The spectrometer was controlled by the developed GUI. The spectral data acquired by the spectrometer was in the spectral range of 400 to 1000 nm. To protect the DRS probe tip from tissue contamination a sterile plastic camera drape (365 Healthcare Ltd.) was chosen due to the low optical interference, low cost, and sterility.

Data were collected from esophageal and/or stomach specimens *ex vivo* immediately after surgical resection. The hand-held DRS probe and tracking system was used on suspected normal tissue within the sample and on macroscopically suspected cancerous or fibrosed tissue to obtain spectral information.

DRS measurements of suspected macroscopic tumor site and normal tissue were taken on the outer (serosal) layer. “Normal” tissue measurements were taken as far from the suspected cancerous location as possible and close to the resection margin, based on visual and haptic inspection of the macroscopic tissue by a surgeon. Twenty spectra were acquired per tissue point, which were averaged and displayed in real time (Fig. 4). A minimum of 200 different locations per tissue type (normal or tumor) were sampled, depending on the size of the region. When possible, DRS recordings of stomach and esophagus were taken from the same specimen. The data acquisition protocol lasted ∼10  min.

To account for inter-patient, background light, and signal quality variability, spectral normalization, and noise reduction was performed at the beginning of each sampling session using white reflectance standard and dark-field readings:

$$\lambda_{\mathrm{DRS}}=\frac{\lambda_{\mathrm{RAW}}-\lambda_{\mathrm{DN}}}{\lambda_{\mathrm{WST}}-\lambda_{\mathrm{DN}}},$$

where $\lambda_{\mathrm{RAW}}$ is the raw signal acquired from the spectrometer, $\lambda_{\mathrm{DN}}$ is the dark-field signal, $\lambda_{\mathrm{WST}}$ is the white reflectance standard signal and $\lambda_{\mathrm{DRS}}$ is the final reflectance value. DRS data were then processed using a Savitzky–Golay (or digital smoothing polynomial) filter to perform noise reduction and preserve higher order moments (spectral characteristics) of the original spectrum. The workflow of the *ex vivo* data acquisition and analysis is shown in Fig. 5(b).

### 2.5 Histopathology Correlation

**Fig. 6:**
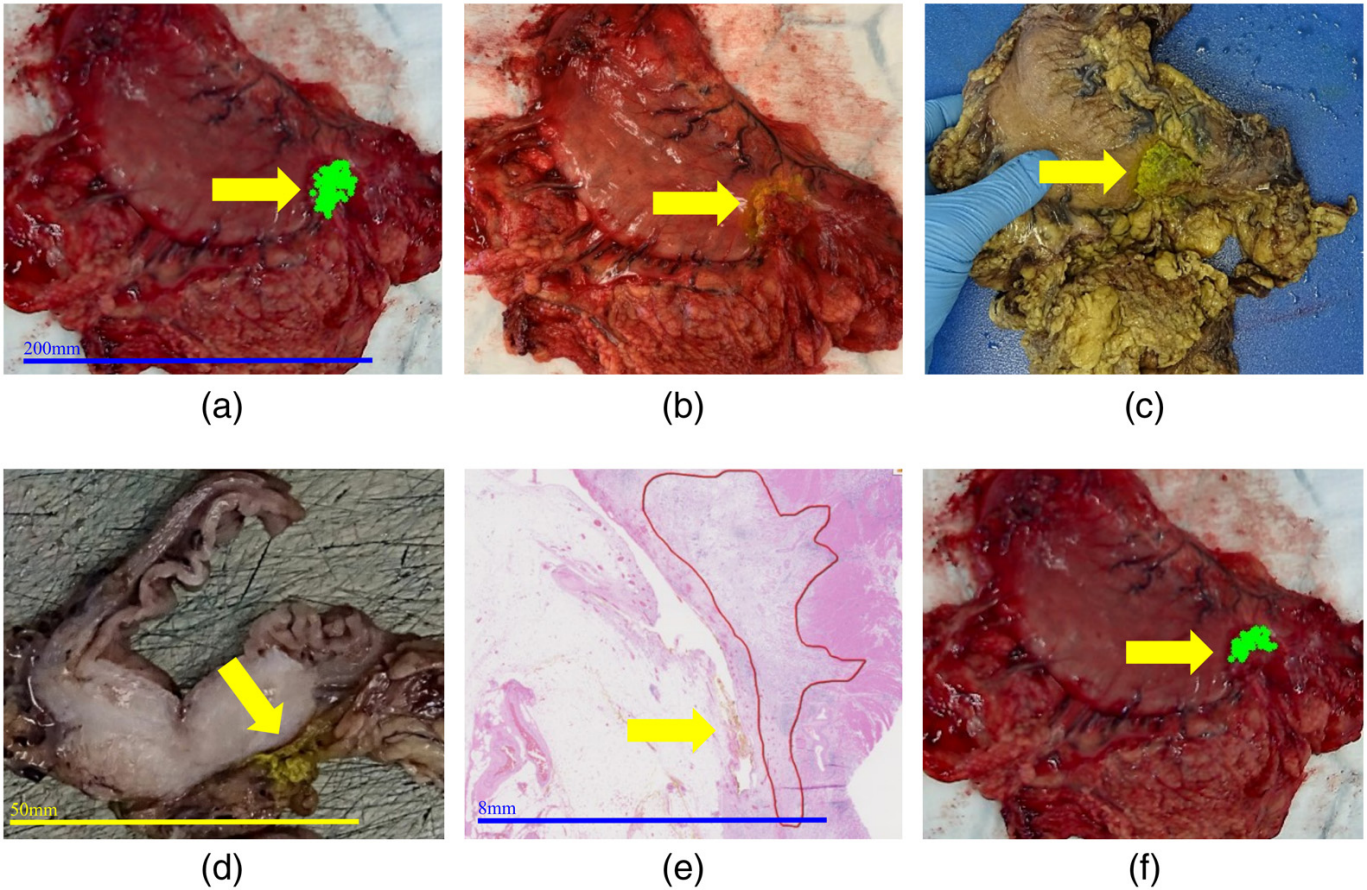
Histopathological analysis of the specimen for purposes of correlation of suspected tumor location. Histopathological correlation workflow is shown on an example of GAC. The correlation was performed via yellow tissue paint [marked with yellow arrow across panels (a)–(f)]. The *ex vivo* tissue specimen (stomach) (a) with tracked optical biopsy sites of the suspected “tumor” area; (b) after painting of macroscopic suspected “tumor” tissue with yellow paint; and (c) having been placed in formalin. (d) Macroscopic slice of stomach tissue. (e) Microscopic H&E slice and confirmed GAC (red outlined area). (f) The *ex vivo* tissue specimen with green colored optical biopsy sites of confirmed tumor [based on (b)] after manual labelling and removal of tracked optical biopsy sites that fell outside of tumor area.

Following the acquisition of all spectra, the suspected tumor location was painted with yellow tissue dye (Cancer Diagnostics Inc., Durham) and another still image was recorded to allow correlation with standard histopathological analysis, which was used as a reference test. The specimen was passed to the histopathology department, where it was sliced and photos were taken of these slices which included the yellow painted region. The tissue was then processed according to standard protocols (Fig. 6), with the resulting histology slides marked as either normal or a range of different tumor types including esophageal adenocarcinoma, esophageal squamous cell carcinoma, or gastric adenocarcinoma.

Once the painted area was confirmed as tumor, the annotated video frames containing the optical biopsy-tracked positions of suspected “tumor” tissue were manually labeled as tumor (Fig. 6). These labels were considered ground truth labels for training of machine learning classifiers. Measurements at locations, which could not be confirmed by histopathology (e.g., if the yellow paint was not clear on the sliced specimen or if the paint could not be detected on the H&E slide) were excluded.

### 2.6 Spectral Classification

**Fig. 7:**
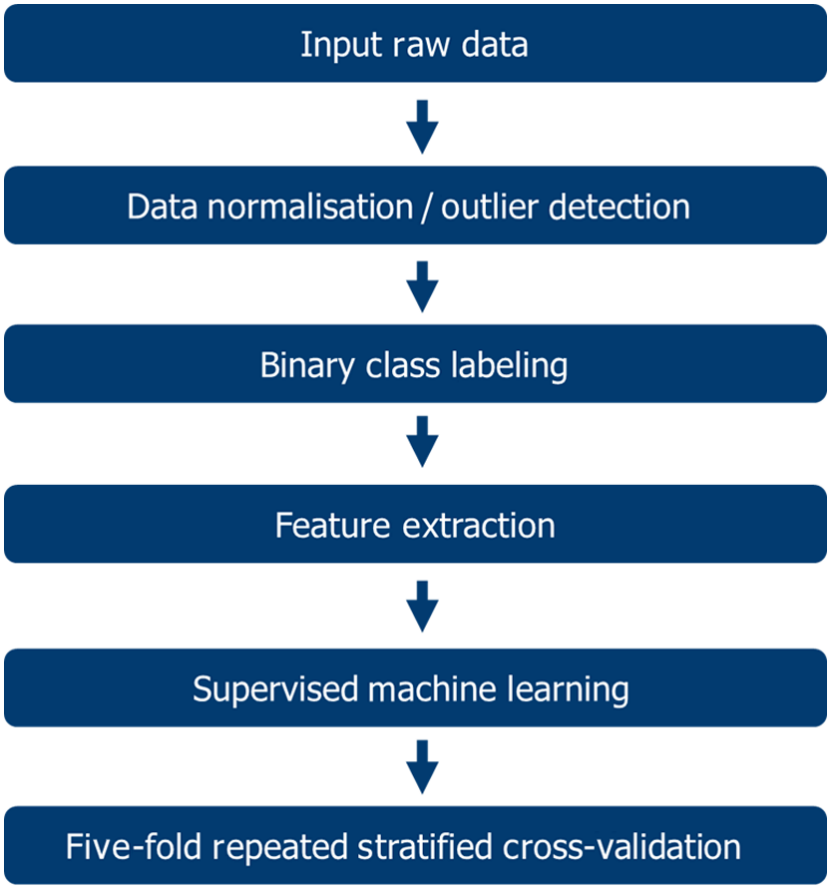
Spectral data analysis workflow.

Data from all patients were separately combined into esophagus and stomach datasets. The features of the collected data were first standardized by removing the mean and scaling to unit variance. An automated method was implemented for outlier (e.g., erroneous measurements or air interference) detection and removal. For this method, the median of the residuals was calculated, along with the 25th and 75th percentiles. The difference between each historical value and the residual mean was then calculated. If the historical value was 1.5 times the median absolute deviation away from the median of the residuals, that value was classified as an outlier. Additionally, the ground truth provided by histopathology correlation was used for the validation of the method. To reduce overfitting and improve accuracy, feature selection was performed on each of the esophagus and stomach datasets. Data were then divided into the training and testing sets using the repeated stratified k-fold cross-validation (CV) method, with five folds and five repeats. This CV method was chosen based on a literature review and the nature of the data used in this study. The stratified five-fold CV method gives a more stable and trustworthy result since training and testing is performed on several different parts of the dataset and deals with imbalanced data. Using this CV method, the dataset is split into five folds such that each fold contains approximately the same percentage of samples of each target class as the complete set. When stratified five-fold CV is compared to leave-one-out CV, the latter requires building n models instead of five, where n stands for the number of samples in the dataset. This means that the leave-one-out CV method is more computationally expensive than the stratified five-fold CV. The spectral data analysis process is shown in Fig. 7.

Binary classification into normal and tumor tissue was performed using various supervised machine learning classifiers, such as linear support vector machine (SVM), multi-layer perceptron (MLP), light gradient boosting machine (LGBM), and extreme gradient boosting (XGB).^26^

Machine learning classifiers were evaluated in terms of sensitivity, specificity, overall accuracy, and the area under the ROC curve (AUC). Python 3.6 was used for data processing, visualization, machine learning classification, and statistical analysis.

## 3 Experimental Evaluation and Results

### 3.1 DRS Probe Tracking Evaluation

**Fig. 8:**
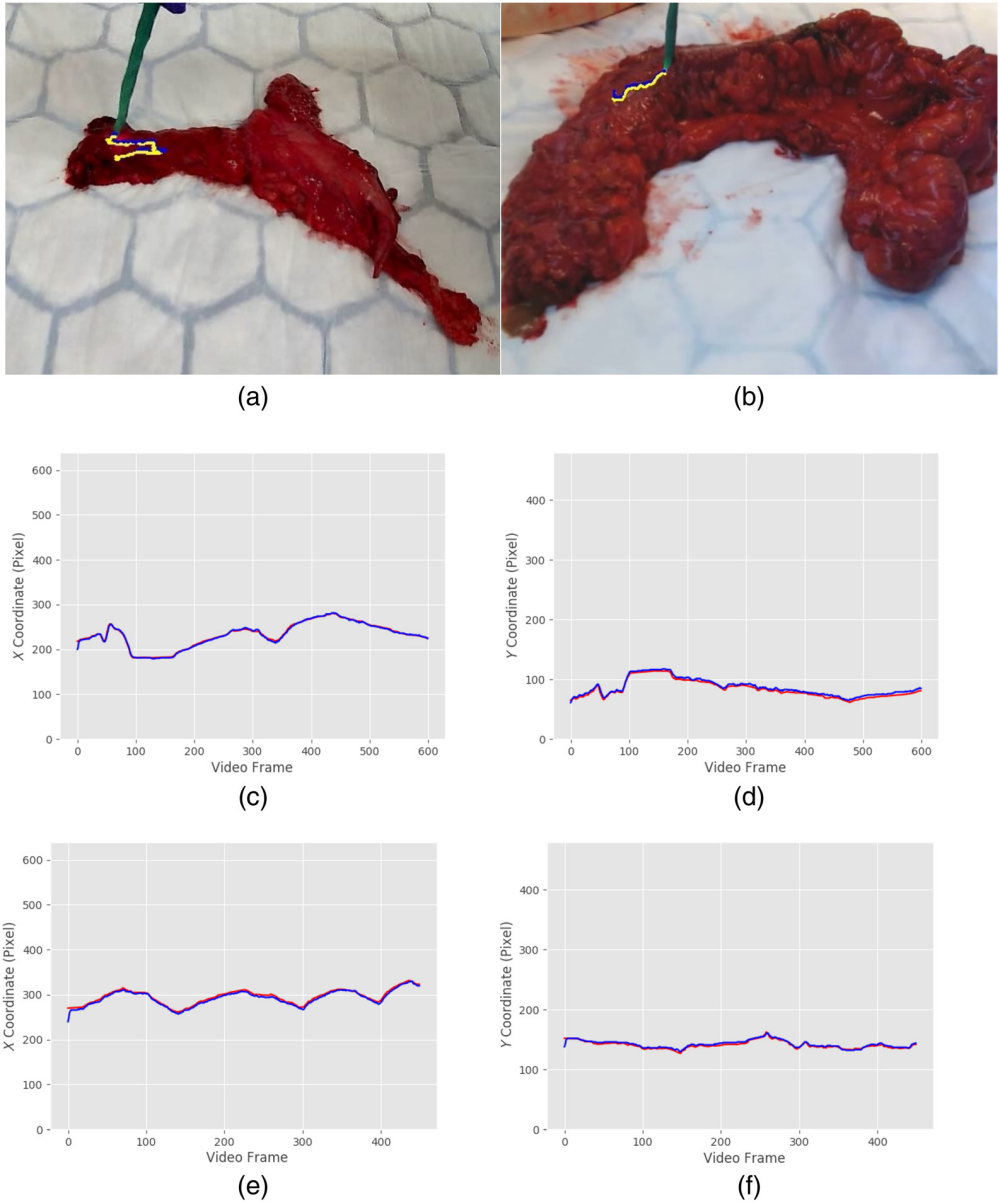
(a) and (b) Illustration of probe tracking error with overlaid ground truth (blue line) and estimated (yellow line) probe tip position for two video sequences (600 and 450 frames each). (c)–(f) The ground truth and estimated projected DRS probe tip pixel position for x axis and y axis for each of the video sequences. Blue and red lines indicate the estimated and ground truth respectively.

For the evaluation of the tracking accuracy, two videos from each tissue specimens were analyzed by manual labeling of the probe 2D tip position (x,y) by two different individuals (data labelling tool Labelbox Inc., California) and consequently compared to the estimated tip location by the proposed algorithm (Fig. 8). The root mean squared error (RMSE) between the estimated and manually labeled tip position was 1.18±0.58  mm (4.21±2.07  pixels) and 1.05±0.28  mm (3.75±0.97  pixels) for the x and y directions, respectively, as shown in Table 1. The maximum measured error across all 1050 frames from both videos was 1.76 mm (6.28 pixels) at a working distance of 30 cm. In Fig. 8, we can see a frame from each video overlaid with the manually labeled and the estimated probe’s tip position in two different colors.

**Table 1:** Projection error for DRS probe’s tip location.

Dimension	RMSE (mm)	Maximum error (mm)	Minimum error (mm)
x	1.18±0.58	1.76	0.00
y	1.05±0.28	1.39	0.00

For further validation, the detection limits and detection rates were calculated by recording the maximal experimentally distance and rotation angle of the probe (Table 2). The distance was recorded from the camera to the probe and the limits of rotation were defined about the probe local coordinate axes (roll, pitch, and yaw). When testing the distance limits, the probe was translated along the axis of the camera until the detection failed. To identify the rotational motion limits, the probe was placed at 30 cm from the camera, a typical distance for practical *ex vivo* tissue scanning.

**Table 2:** Rotation angle and maximum detectable distance from the camera to track the DRS probe.

Rotation axis	Rotation angle and distance
Roll (deg)	360 deg
Pitch (deg)	360 deg
Yaw (deg)	0 deg to 180 deg
Distance to camera (cm)	10 to 100

The image-based tracking framework was used to map all spatio-temporally tracked biopsy sites and re-project them back onto the image plane to provide a live augmented view of the *ex vivo* spectral acquisition procedure. The biopsy site was defined by the probe tip location, taken from the tracking procedure, and the geometrical characteristics of the probe.

### 3.2 Real-time Tracking and Sampling of *Ex Vivo* Tissue

A total of 32 patients were recruited into this study. Seven patients were excluded due to histopathology assessment, as tumor was reported benign or regressed, resulting in 26 patients being included in the final analysis. The median age was 68, with the majority of patients being male (n=24, 75%). Overall, 20 distinct sets of normal stomach data, 12 of normal esophagus data, 8 of gastric cancer data, and seven of esophageal cancer data were recorded. *Ex vivo* specimen labelling errors were identified by reviewing videos of real-time data acquisition retrospectively and analyzing the DRS probe position over the tissue areas through use of the color tracking system. Probe contact artifacts (e.g., lack of probe contact with the tissue) were removed using the automated outlier detection and removal method mentioned in Sec. 2.6.

**Fig. 9:**
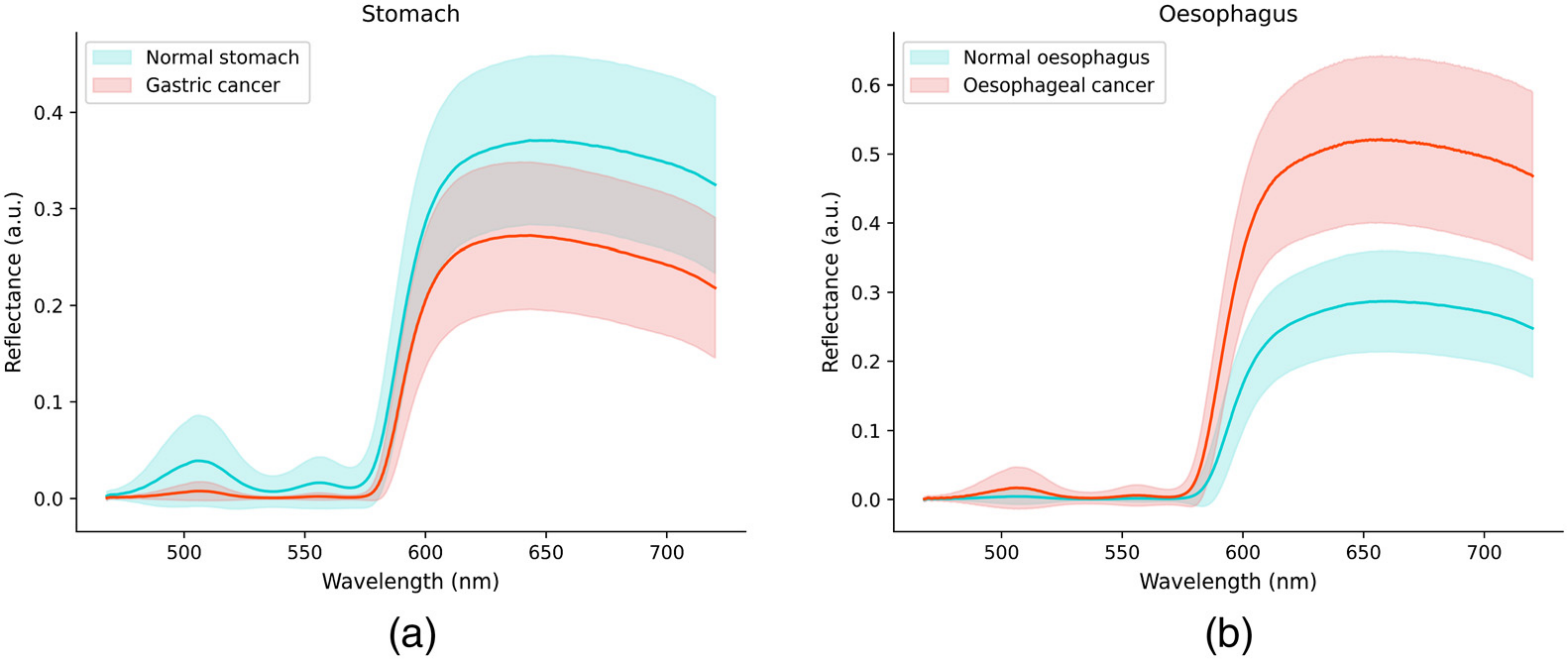
Average (lines) and standard deviations (shaded areas) of the mean spectra for (a) stomach and (b) esophagus.

A total of 4628 mean spectra were collected for normal stomach, 2305 mean spectra for gastric cancer, 2990 mean spectra for normal esophagus, and 1939 mean spectra for esophageal cancer. Each processed mean spectrum contained 505 equally spaced intensity measurements in the 468- to 720-nm spectral range (resolution 0.5 nm) with data from 420 to 468 nm, and 720- to 1000-nm spectral ranges excluded following interim analysis. The means of all spectra for each of the tissue classes are shown in Fig. 9.

### 3.3 Classification of Tumor versus Non-Tumor

Results of the classification for stomach and esophagus spectral data is presented in Table 3. XGB was the best performing machine learning algorithm for both stomach and esophagus, achieving an overall normal versus cancer diagnostic accuracy of 93.86±0.66 for stomach and 96.22±0.50 for esophagus. The sensitivity and specificity of the classifier were 91.31% and 95.13% for stomach and 94.60% and 97.28% for esophagus. For the XGB algorithm, a step size shrinkage of 0.3 was used in the update phase to prevent overfitting, while the maximum depth of a tree was chosen at value of 6 as a higher value of this parameter would make the model more complex and more likely to overfit. The XGB and LGBM algorithms are both ensemble tree methods that apply the principle of boosting weak learners using the gradient descent architecture. However, XGB improves upon the base gradient boosting machines framework through parallelization of tree building, tree pruning using depth-first approach, regularization for avoiding overfitting, and in-built CV capability. Compared with the SVM model, the XGB algorithm generally showed better performance in terms of accuracy, sensitivity, and specificity. In addition, the XGB model showed much higher computation speed than all the other algorithms used in this study, due to its inherent parallel processing, requiring only 3.5 s over both training and validation phases.

**Table 3:** Performance metrics for the spectral data classification using XGB. Data presented as mean ± standard deviation. Overall accuracy calculated as proportion of correctly identified spectra over total number of spectra.

Tissue type	Classifier	Accuracy (%)	Sensitivity (%)	Specificity (%)	AUC (%)
Stomach	XGB	93.86±0.66	91.31±1.59	95.13±0.84	98.50±0.28
Stomach	LGBM	93.63±0.72	91.00±1.66	94.94±0.79	98.40±0.28
Stomach	MLP	90.06±0.96	86.27±4.40	91.74±1.41	96.09±0.50
Stomach	SVM	87.67±0.81	83.47±1.27	89.53±1.05	94.23±0.52
Esophagus	XGB	96.22±0.50	94.60±0.92	97.28±0.63	99.24±0.19
Esophagus	LGBM	96.26±26	94.38±0.98	97.47±0.57	99.27±0.18
Esophagus	MLP	91.97±2.90	85.18±4.09	95.97±2.90	96.74±0.45
Esophagus	SVM	88.35±0.95	82.19±4.30	92.34±2.33	94.19±0.53

**Fig. 10:**
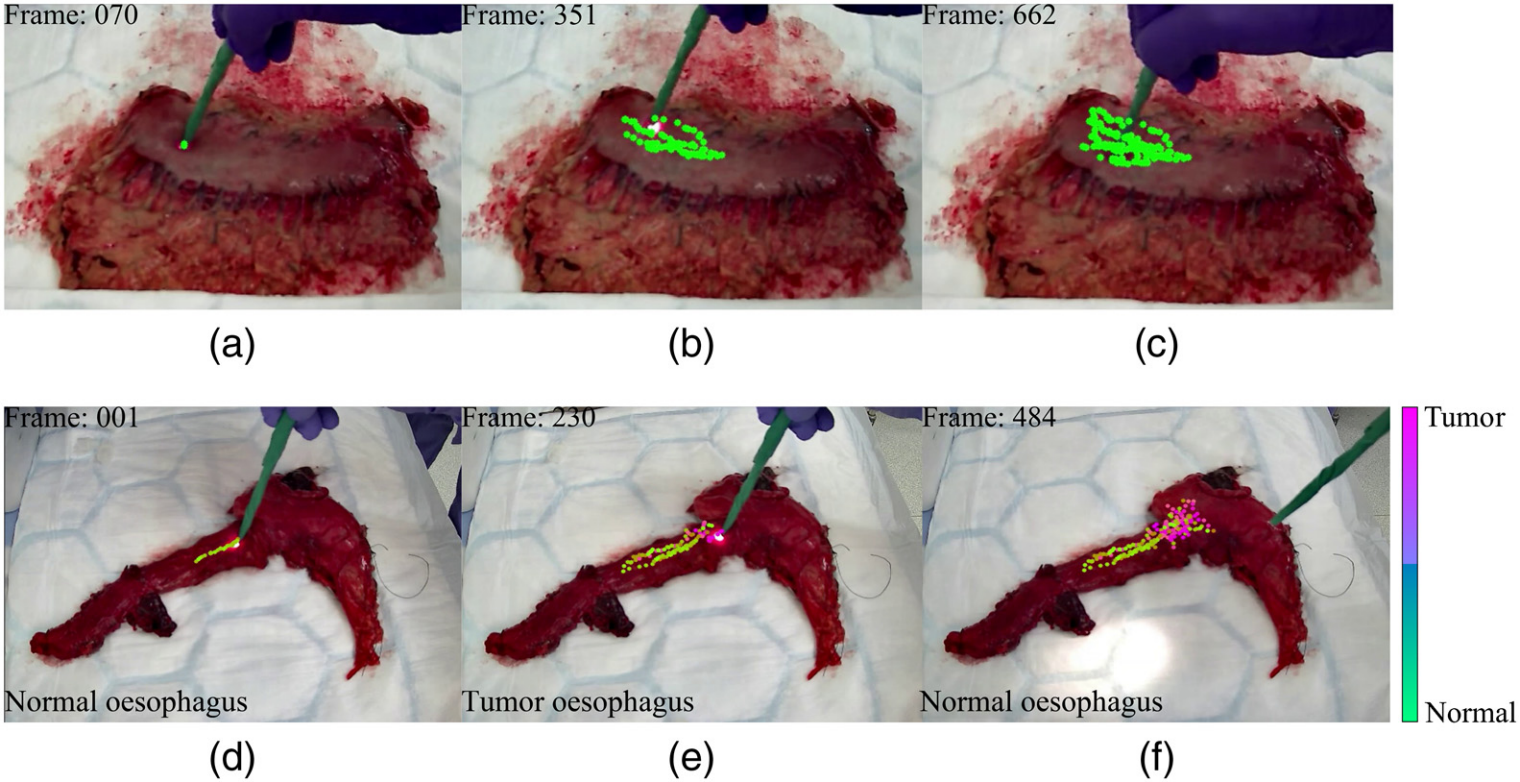
Illustration of real-time DRS probe tracking and classification at different frames during scanning of a gastric *ex vivo* tissue specimen. (a)–(c) Real-time visual correlation of scanned optical biopsy sites on tissue (green dots on each frame) using DRS probe. (d)–(f) Real-time tracking at each optical biopsy site coupled with binary classification of each site as either normal (100% green) or tumor tissue (100% pink) via a graduated color map. Tissue type highlighted on screen in real time (bottom left of each frame).

## 4 Discussion

We have successfully developed a real-time tracking system for a DRS probe when used on stomach and esophageal tissue for tumor detection. Our tracking system showed a measured root mean square error of 1.18 and 1.05 mm for the X and Y directions, respectively, and a maximum measured error of 1.76 mm when used. The color green achieved an excellent performance when used as a marker for probe tracking. This tracking system allowed us to classify spectral data for tumor and non-tumor tissue in real-time with an overall diagnostic accuracy of 94% for stomach and 96% for the esophagus, highlighting the clinical value of our technique.

Our handheld DRS probe and tracking system was able to acquire 80 spectra per second while providing real-time diagnostic information through direct visualization of the areas on the specimen that were probed (Fig. 10). Probe tracking was performed in real time at 30 frames per second.

Different types of optical spectroscopy techniques (e.g., Raman spectroscopy, fluorescence, and DRS) are being investigated for sensing tissue types.^27^ Diffuse reflectance is well suited to real-time characterization of tissue properties and acquisition of information in a short space of time together with ease of use.^22^^,^^28^^–^^30^ The real-time tracking method we have developed in this study, and the technique for accurate classification of tumor tissue, can also be applied to other optical spectroscopy probes, such as rapid evaporative ionization mass spectrometry (REIMS) technology,^31^^,^^32^ fluorescence spectroscopy^33^ and Raman spectroscopy.^34^^,^^35^ In this way, the ergonomics, ease of use and validation of data collection for these optical techniques can be improved.

In our study, we were able to probe the *ex vivo* specimen within 15 min of tissue resection, with the real-time spectral data acquisition protocol lasting ∼10  min. This meant that the tissue was probed as close to its *in vivo* setting as possible and ensured minimal disruption to the specimen handling and processing.

There are a number of limitations for this study. First, this is an *ex vivo* study in which specimens were placed on a white background and colors in the surrounding field could be controlled. However, application *in vivo* may not allow this and can introduce differences in color and artefacts (e.g., active bleeding and other instruments), leading to difficulties in tracking of the color marker. Second, the accuracy of the tracking method could be further increased by the use of modern deep learning models for probe tip detection and tracking.^36^^–^^39^ Third, the tracking system used in this study provided two-dimensional (2D) visual information only, although we are currently collecting three-dimensional (3D) stereoscopic data to aid clinical application in the future.^40^^,^^41^ We are also concurrently developing further classification statistics based on benign or regressed tumor to increase the clinical value of the system, since this study only focused on tumor and non-tumor tissue. Finally, in laparoscopic surgery, probe tracking will have to occur with a moving laparoscopic camera.^42^^,^^43^ Moreover, occlusions of the probe caused by other instruments and the tissue will provide additional optical tracking challenges.

## 5 Conclusion

The proposed real-time DRS tracking method has been validated on *ex vivo* data with histological ground truth, and the accuracy derived demonstrates the strength and clinical value of the technique. The method allows real-time tracking and accurate classification with short data acquisition time to aid margin assessment in cancer resection surgery and has potential to be applied in routine surgical practice. The methods developed can provide an aid for tissue tracking and validation against other diagnostic methods, improving the process of evaluating new spectroscopy modalities.

## Code, Data, and Materials Availability

Raw data are available upon reasonable request.
